# Interleukin-23 Represses the Level of Cell Senescence Induced by the Androgen Receptor Antagonists Enzalutamide and Darolutamide in Castration-Resistant Prostate Cancer Cells

**DOI:** 10.1007/s12672-020-00391-5

**Published:** 2020-06-20

**Authors:** Siddharth Gupta, Thanakorn Pungsrinont, Ondrej Ženata, Laura Neubert, Radim Vrzal, Aria Baniahmad

**Affiliations:** 1 Institute of Human Genetics, Jena University Hospital, Am Klinikum 1, 07740 Jena, Germany; 2 Department of Cell Biology and Genetics, Palacky University, Šlechtitelů 27, 78371 Olomouc, Czech Republic

**Keywords:** Cellular senescence, Androgen receptor antagonists, Interleukin-23, Prostate cancer spheroids

## Abstract

Prostate cancer (PCa) is the most common cancer and the second leading cause of cancer-related deaths of men in Western countries. Androgen deprivation therapy is initially successful, however eventually fails, and tumors progress to the more aggressive castration-resistant PCa (CRPC). Yet, androgen receptor (AR) usually remains as a major regulator of tumor cell proliferation in CRPC. Interleukin-23 (IL-23) was recently shown to promote the development of CRPC by driving AR transcription. Here we used the androgen-sensitive LNCaP, castration-resistant C4-2, and 22Rv1 cells. Interestingly, cellular senescence is induced in these human cell lines by treatment with the AR antagonists enzalutamide (ENZ) or darolutamide (ODM), which might be one underlying mechanism for inhibition of PCa cell proliferation. Treatment with IL-23 alone did not change cellular senescence levels in these cell lines, whereas IL-23 inhibited significantly cellular senescence levels induced by ENZ or ODM in both CRPC cell lines C4-2 and 22Rv1 but not in LNCaP cells. This indicates a response of IL-23 specific in CRPC cells. Generating LNCaP and C4-2 three-dimensional (3D) spheroids and treatment with AR antagonists resulted in the reduced spheroid volume and thus growth inhibition. However, the combination of AR antagonists with IL-23 did not affect the antagonist-mediated reduction of spheroid volumes. This observation was confirmed with proliferation assays using adherent monolayer cell cultures. Taken together, the data indicate that IL-23 treatment reduces the AR antagonists-induced level of cellular senescence of CRPC cells, which could be one possible mechanism for promoting castration resistance.

**Electronic supplementary material:**

The online version of this article (10.1007/s12672-020-00391-5) contains supplementary material, which is available to authorized users.

## Introduction

Prostate cancer (PCa) is the most common cancer and the second leading cause of cancer-related deaths of men in Western countries [1]. PCa growth depends on androgens at initial phases, and therefore, androgen ablation became a major form of PCa therapy. Androgens exert their role via the ligand-controlled transcription factor androgen receptor (AR). Therefore, inhibition of the AR signaling represents a major drug target in the treatment of PCa. Despite the first line of therapy being successful, the cancer eventually develops to the castration-resistant PCa (CRPC) form. Interestingly, the AR signaling usually remains still active in CRPC but shows an adaptive AR signaling response [2, 3]. Thus, inhibition of AR-mediated transactivation by second-generation antagonists such as by enzalutamide (MDV3100; ENZ) is successful and one of the major hormone therapy option for CRPC. Darolutamide (ODM-201; ODM) is also a novel AR antagonist with a distinct chemical structure compared with other AR antagonists and currently in clinical phase 3 trials for CRPC [4].

Mechanistically, we have previously shown that AR antagonists such as the nonsteroidal atraric acid [5] and compound C28 [6, 7] induce cellular senescence in PCa cells. Cellular senescent cells are arrested irreversibly in cell cycle and thus suggested that this pathway is a tumor-suppressive pathway [8, 9].

In addition to intrinsic adaptive signaling of PCa cells, external factors may also influence castration resistance. IL-23 produced by myeloid-derived suppressor cells (MDSCs) was shown to serve as promoter of CRPC [10]. Interestingly, IL-23 activates the AR signaling and enhances cell proliferation in a non-cell autonomous manner in PCa cells [10].

Therefore, we hypothesize that IL-23 interferes with AR antagonist-induced cellular senescence. We show that ENZ and ODM reduce growth of 2D monolayer and in 3D spheroid volume of both LNCaP and C4-2 cells and induce cellular senescence. Interestingly, antagonist-induced cellular senescence is counteracted by IL-23 treatment in C4-2 and 22Rv1 cells, both human CRPC cell lines, indicating that IL-23 interferes with AR signaling-mediated cellular senescence in PCa.

## Materials and Methods

### Cell Lines and Culture

The androgen-dependent human PCa cell line LNCaP [11] and the human CRPC cell line 22Rv1 (ECACC 05092802; Public Health England) were cultured in RPMI 1640 medium (Gibco Life Technologies) supplemented with 5% FBS (Gibco Life Technologies), 1% penicillin/streptomycin (Gibco Life Technologies), 1% sodium pyruvate (Gibco Life Technologies), and 25 mM HEPES (pH 7.5). The CRPC cell line C4-2, which is derived from LNCaP cells [12], was cultured in DMEM supplemented with D-glucose (4.5 g/L)/L-glutamine/pyruvate (Gibco Life Technologies) as well as 20% F-12 nutrient mixture (Gibco Life Technologies), 5% FBS, 1% penicillin/streptomycin, and 25 mM HEPES pH 7.5. All three cell lines were cultivated on 10 cm cell culture dishes at 37 °C, 5% CO_2_ humidified atmosphere.

Forty-eight hours after seeding for monolayer experiments, one set of LNCaP, 22Rv1, and C4-2 cells was pretreated with 100 ng/ml recombinant human IL-23 (rIL-23) (PeproTech) for 1 h followed by cotreatment with 0.1% DMSO (control), 10 μM ENZ (Selleck Chemicals), or 10 μM ODM (Selleck Chemicals) for further 72 h. The other set of cells was treated with DMSO and AR ligands without pretreatment of rIL-23. In each experiment, DMSO and AR antagonist treatment was performed in duplicates.

### 3D Spheroid Assays

Cells were harvested from 80% confluent 10 cm cell culture dish and seeded at 1000 cells/well in 96-well ultralow attachment plates (PerkinElmer). The cells were centrifuged at 300 rpm, 3 min at room temperature (RT), and incubated at 37 °C, 5% CO_2_ to form a spheroid. After 72 h of spheroids formation (day 0), one set of the spheroids was pretreated with 100 ng/ml rIL-23 for 6 h followed by treatment with 0.1% DMSO, 10 μM ENZ, or 10 μM ODM. The other set was treated with DMSO and AR antagonists without pretreatment of rIL-23. The imaging of spheroids by brightfield microscope CellObserverZ1 (Carl Zeiss; objective, 10x/0.3 Plan-Neofluar; Camera, AxioCam MRm R3-12bit monochrome camera 1388 × 1040 pixel) as well as the re-treatment of AR ligands were performed every 48 h. The resulting images were processed using ZEN Blue software (Carl Zeiss), and spheroid’s size was measured by Fiji ImageJ software. The formula for the spheroid volume = ^4^/_3_(π*r*^3^), where *r* is geometric mean radius. The formula for the geometric mean radius = ½(*a* × *b*)^1/2^, where *a* and *b* are the two orthogonal diameters of the spheroid as described in Puhr et al. [13]. Three independent experiments were performed with each treatment.

### Crystal Violet Staining

For growth assays, LNCaP, C4-2, and 22RV1 cells were seeded in 6-well tissue culture plates (Greiner Bio-One International) at 1.3 × 10^4^ cells per well. To analyze the effect of treatments on PCa cell growth, the crystal violet staining was performed as described earlier [14, 15] as an indirect measurement of cell number at day 3 and day 6 of incubation. The crystal violet stain of cells was solubilized with Sørenson’s solution as described previously [16]. The absorbance was measured at 590 nm using UV/Vis spectrophotometer. Two wells per experiment were measured and experiments were performed three times.

### Senescence Associated β-Galactosidase (SA-β-Gal) Staining

For cellular senescence assays, cells were seeded in 6-well tissue culture plates at 5 × 10^4^ cells per well. To analyze the effect of treatments on cellular senescence induction, the SA-β-Gal staining was performed after 3 days of treatment with the indicated compounds as described earlier [17, 18]. The stained cells were detected and counted by light microscopy. Six random fields per treatment were selected and at least 200 cells per field were counted. Three independent experiments were performed. The percentage of stained cells was then determined and calculated as fold induction in relative to control treatment.

### Quantitative Reverse Transcription Real-Time PCR (RT-qPCR)

Cells were seeded in 10 cm cell culture dishes at 5 × 10^5^ cells per dish. To detect senescence-associated changes of cell cycle inhibitors, total RNA extraction was performed using peqGOLD TriFast™ reagent (Peqlab) according to the manufacturer’s protocol. Two-step RT-qPCR was conducted as earlier described [14, 15]. Briefly, the cDNA was first synthesized using the High Capacity cDNA Reverse Transcription kit (Applied Biosystems). The PCR was performed using SsoAdvanced Universal SYBR Green Supermix (Bio-Rad), gene specific primers, and Bio-Rad CFX96TM real-time PCR (RT-qPCR) detection system with 4 technical replicates normalized to *TBP* mRNA. The primer sequences are listed as 5′ → 3′:

- *TBP*: fwd: GGCGTGTGAAGATAACCCAAGG
  rev: CGCTGGAACTCGTCTCACT
- *KLK3*: fwd: GAGGCTGGGAGTGCGAGAAG
  rev: TTGTTCCTGATGCAGTGGGC
- *FKBP5*:fwd: GAGGAAACGCCGATGATTGGAGAC
  rev: CATGCCTTGATGACTTGGCCTTTG
- *CDKN2A*: fwd: CTTGCCTGGAAAGATACCG
  rev: CCCTCCTCTTTCTTCCTCC

### Antibodies and Western Blot Analysis

Cells were seeded in 10 cm cell culture dishes at 5 × 10^5^ cells per dish. After 72 h of AR ligand treatment with or without IL-23 treatment, protein extraction from the whole-cell lysates, Western blotting, and quantification of proteins were performed as earlier described [14]. Primary and secondary antibodies for p16, α-Tubulin, and β-Actin detection were previously described [14, 18]. For other targets, the primary antibodies used for immunodetection were IL-23R (Novus Biologicals, NB600-1147SS) detecting three isoforms, STAT3 (Cell Signaling, 9132), and phosphorylated STAT3 (Cell Signaling, 9145).

### Statistical Analysis

Two-tailed unpaired Student’s *t* test and two-way ANOVA were performed for differential comparison between two groups using GraphPad Prism 8.0 software. A value of *p* < 0.05 was considered statistically significant.

## Results

### ENZ and ODM Reduce Growth and Induce Cellular Senescence of Androgen-Sensitive LNCaP and Castration-Resistant C4-2 and 22Rv1 Cells

**Fig. 1:**
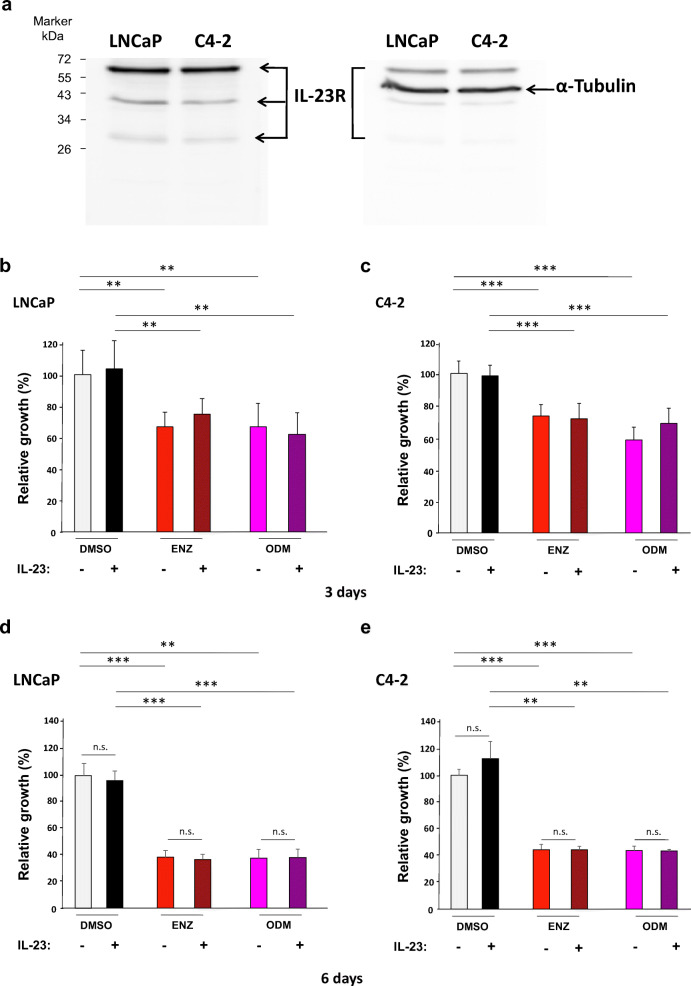
ENZ and ODM inhibit proliferation of LNCaP and C4-2 cells. **a** Western blotting analysis reveals that IL-23 receptor (IL-23R) is expressed in both androgen-dependent LNCaP and castration-resistant C4-2 cells detecting three isoforms. α-Tubulin serves as loading control on the same membrane. **b**–**e** Growth assays were performed with LNCaP and C4-2 cells. Cells were treated for 3 days (b, c) or for 6 days (d, e) in the presence of ENZ (10 μM) or ODM (10 μM) in the absence or presence of 100 ng/ml recombinant human IL-23. Proliferation was analyzed by crystal violet staining with subsequent measurement of absorbance at 590 nm. As solvent control, DMSO was used. *n* = 3; *, *p* < 0.05; **, *p* < 0.01; ***, *p* < 0.001

To analyze the effect of IL-23, first the presence of IL-23 receptor (IL-23R) was confirmed by Western blotting in both LNCaP and C4-2 cells (Fig. 1a). The antibody recognizes three isoforms with the amino acid length of 629, 390, and 227. It was shown that IL-23 regulates STAT3 phosphorylation [10]. In order to test IL23R functionality, STAT3 phospho-status was measured accordingly. We found enhanced phospho-STAT3 (p-STAT3) levels in C4-2 while less pronounced in LNCaP cells by IL23 treatment (Fig. S1). This indicates that STAT3 signaling is more responsive to IL-23 in the CRPC C4-2 cells. To analyze cell growth by the two AR antagonists, ENZ and ODM, on androgen-sensitive LNCaP and the castration-resistant C4-2 cell lines, ligand treatment was performed for 3 and 6 days. In addition, we pretreated cells with and without IL-23 for an hour and cotreated IL-23 with antagonist treatment for additional 72 h. As expected, both AR antagonists reduced the proliferation of three PCa cell lines significantly at day 3 (Fig. 1b, c and Fig. S2 for 22Rv1 cell line) and more pronounced when treating longer for 6 days (Fig. 1e, f). The treatment with IL-23 did not reveal a significant alteration of proliferation within the tested time period.

### IL-23 Alters AR Antagonist-Induced Cellular Senescence

**Fig. 2:**
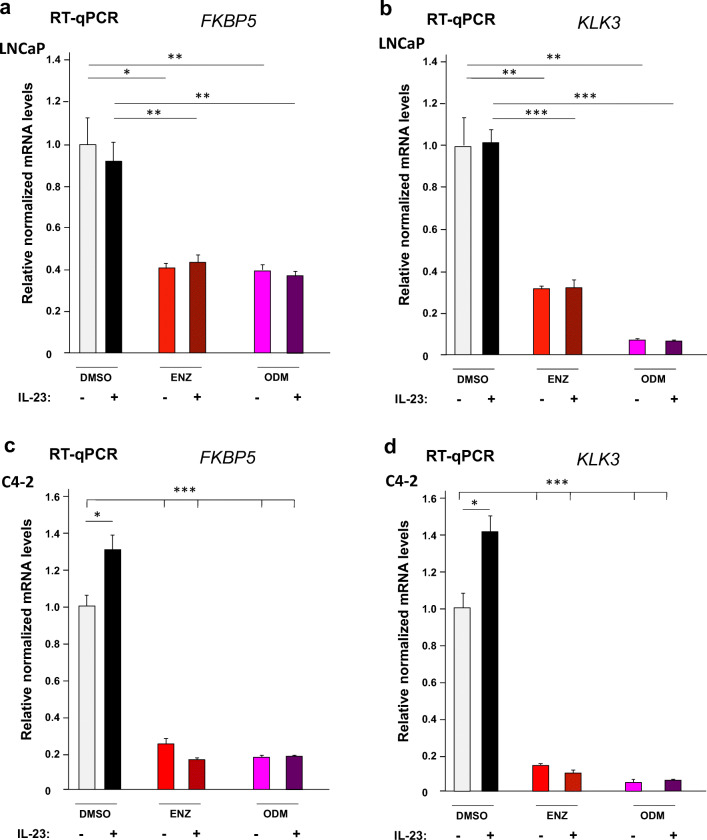
ENZ and ODM inhibit the AR target genes *KLK3* and *FKBP5* in both LNCaP and C4-2 cells. LNCaP and C4-2 cells were treated with or without IL-23 (100 ng/ml) for an hour prior the treatment of indicated compounds DMSO (0.1%), ENZ (10 μM), and ODM (10 μM) for further 72 h. RNA was extracted and subjected to RT-qPCR. As housekeeping gene, *TBP* was used for normalization of the mRNA levels of LNCaP cell’s (**a**) *FKBP5*, (**b**) *KLK3*, and C4-2 cell’s (**c**) *FKBP5* and (**d**) *KLK3*. The DMSO control was set arbitrarily as 1 and the obtained values are depicted relative to DMSO control. *n* = 4; *, *p* < 0.05; **, *p* < 0.01; ***, *p* < 0.001

To analyze whether IL-23 interferes with AR-mediated gene regulation, the expression of endogenous AR target genes *KLK3* encoding PSA and *FKBP5* were analyzed by RT-qPCR using both LNCaP and C4-2 cells in the presence or absence of the AR antagonists and IL-23. The data suggest that both AR antagonists repress the expression of *FKBP5* and *KLK3* (Fig. 2). Interestingly, ODM represses more potently *KLK3* mRNA levels compared with ENZ, whereas *FKBP5* is repressed to a similar level. This effect is observed in both LNCaP and C4-2 cell lines. Treatment with IL-23 alone had no significant effect on these AR target genes in LNCaP cells (Fig. 2a, b) and slightly but significantly enhanced the mRNA levels of both AR target genes in C4-2 cells (Fig. 2c, d). Cotreatment of IL-23 with the AR antagonists had no effects on the AR target gene expression (Fig. 2).

**Fig. 3:**
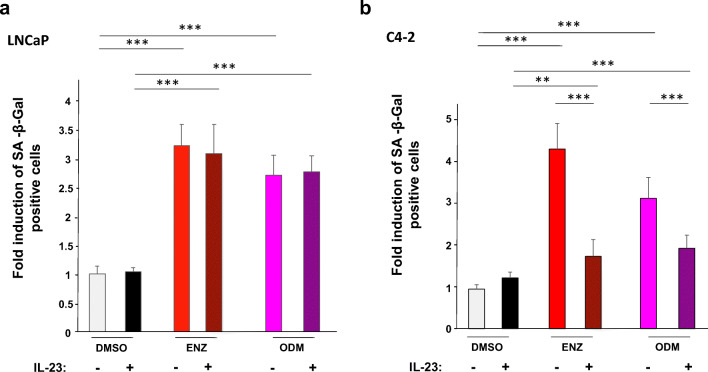
IL-23 inhibits ENZ- and ODM-induced cellular senescence in C4-2 cells but not in LNCaP. The experiments were performed as described in Fig. 2 with **a** LNCaP and **b** C4-2 cells. After 72 h of AR ligand treatment, the cellular senescence specific marker SA-β-Gal was analyzed. Six random fields per treatment were selected, and at least 200 cells per field were counted for each experiment. Three independent experiments were performed. *, *p* < 0.05; **, *p* < 0.01; ***, *p* < 0.001

Since we found that the AR antagonists atraric acid and C28 induced cellular senescence in LNCaP cells [5, 6], we analyzed whether ENZ and ODM are also capable to induce cellular senescence in these cells. Using the SA-β-Gal as a senescence-specific marker, a robust and significant induction of cellular senescence level was observed in both LNCaP and C4-2 cell lines by both AR antagonists (Fig. 3a, b). Also in 22Rv1 cells, either ENZ or ODM treatment induced cellular senescence (Fig. S3).

Treatment with IL-23 alone did not change the basal senescence levels in both LNCaP and C4-2 cell lines (Fig. 3). However interestingly, a combinatory treatment of AR antagonists with IL-23 significantly reduced the AR antagonist-induced level of SA-β-Gal-stained cells in both CRPC cell lines (Fig. 3b and Fig. S3) but not in LNCaP cells (Fig. 3a). This suggests that IL-23 counteracts ENZ- and ODM-mediated induction of cellular senescence in CRPC cells.

**Fig. 4:**
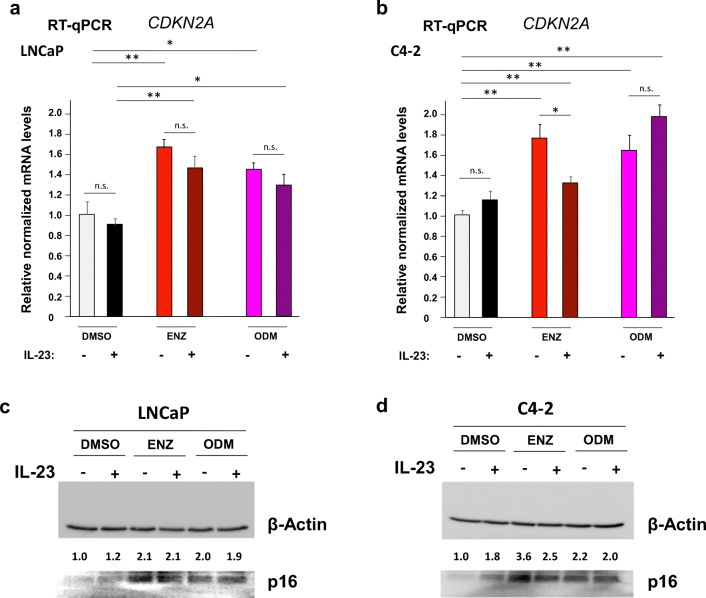
IL23 reduces ENZ-induced *CDKN2A* mRNA levels in C4-2 cells. The experiments were performed as described in Fig. 2. mRNA levels of *CDKN2A* encoding p16 were analyzed from **a** LNCaP and **b** C4–2 extracts by RT-qPCR. Protein levels of p16 of **c** LNCaP and **d** C4-2 cells were analyzed by Western blotting and normalized to β-Actin levels. The DMSO control was set arbitrarily as 1 and the obtained values are depicted relative to DMSO control

Since we identified p16 as a mediator of AR-induced cellular senescence [5, 18], we analyzed the expression of *CDKN2A* encoding p16 (Fig. 4). The increased *CDKN2A* mRNA by ENZ and ODM treatment is in line with our previous findings that the AR-mediated cellular senescence is associated and in part mediated by induction of p16 [18]. Treatment with IL-23 alone did not significantly change the basal levels of *CDKN2A* mRNA in LNCaP or C4-2 cells (Fig. 4a, b). In LNCaP cells, the mRNA level of *CDKN2A* was not changed significantly by IL-23 in the presence of AR antagonists. However, cotreatment of ENZ with IL-23 significantly reduced the levels of *CDKN2A* mRNA levels in C4-2 cells. Western blotting data reveal that p16 is induced by both AR antagonists in both cell lines (Fig. 4c, d). In contrast to C4-2 cells, cotreatment of AR-antagonists with IL-23 does not seem to change the p16 protein level in LNCaP cells (Fig. 4c). However, cotreatment of AR antagonists with IL-23 reduces the p16 levels more potently for ENZ than ODM in C4-2 cells (Fig. 4d). This suggests that IL-23 reduces ENZ-induced cellular senescence via reduction in *CDKN2A* mRNA and p16 protein level.

Taken together, IL-23 reduces the level of cellular senescence induced by treatment with the AR antagonists ENZ or ODM in the CRPC cell line models C4-2 and 22Rv1.

### ENZ and ODM Reduce the Size of PCa Tumor Spheroids

**Fig. 5:**
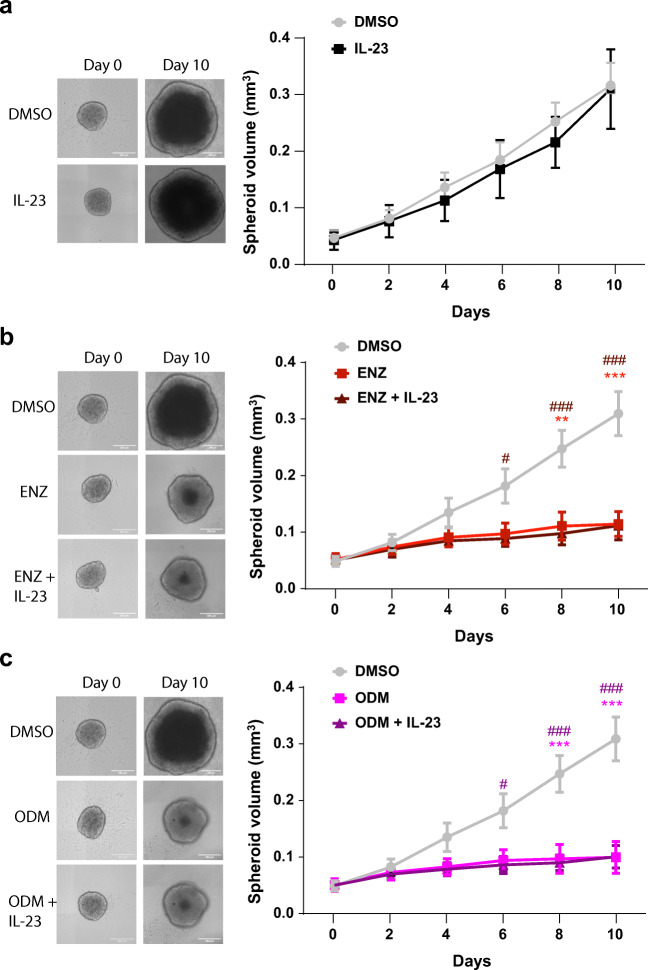
ENZ and ODM reduce potently growth of 3D spheroids. Spheroids were performed using ultralow attachment plates with C4-2 cells. Spheroids were treated for 10 days with the indicated compounds **a** IL-23 (100 ng/ml), **b** ENZ (10 μM), and **c** ODM (10 μM) in combination with or without IL-23 treatment. DMSO was used as a solvent control. Left panel indicates the representative spheroids, and the right panel plots the spheroid volume of three independent experiments at each time points. Asterisk indicates the statistical significance of the AR antagonist compared with DMSO and number sign with and without IL-23 treatment. *, *p* < 0.05; **, *p* < 0.01; ***, *p* < 0.001

Further, we focused on C4-2 cells and generated 3-dimensional (3D) spheroids that were formed using ultralow attachment plates. 3D spheroids are suggested to mimic a tumor in terms of complexity and drug delivery compared with monolayer cultures [19]. Spheroids were successfully generated from C4-2 cells (Fig. 5) but were not properly formed by 22Rv1 (Fig. S4). The C4-2 spheroids were treated with either ENZ or ODM with and without IL-23 for 10 days. IL-23 alone did not affect the spheroid growth (Fig. 5a).

Treatment with either ENZ or ODM potently reduced C4-2-derived spheroid volume (Fig. 5b, c and Fig. S5) suggesting that both AR antagonists inhibit cell proliferation in the 3D spheroid tumor model. Cotreatment of AR antagonists with IL-23 had no effect on spheroid size (Fig. 5b, c and Fig. S5).

## Discussion

The challenging part of PCa therapy is the emergence of CRPC with an active AR signaling [2]. Therefore, the development of new generation of antagonists that inhibit the AR signaling in CRPC represents a promising therapeutic strategy. ENZ as a second-generation AR antagonist is used to treat CRPC; however, therapy resistance to ENZ is still a major concern [10, 20]. Recently, it has been shown that IL-23 secreted from MDSCs promotes castration resistance. Also, recombinant IL-23 promoted markers and characteristics of CRPC [10]. Hence, to build a progress in PCa therapy, it is essential to understand the detailed mechanisms accounting for anti-androgenic activity and resistance to AR antagonists and consequently to develop other strategies to overcome tumor growth progression.

In the present study, we demonstrated that ENZ and ODM repressed the proliferation of PCa cells, which can be explained by the induction of cellular senescence. To our knowledge, this is the first report revealing that treatment with ODM can induce SA-β-Gal activity. In line with this, we observed an induction of the cell cycle inhibitor *CDKN2A* (p16) considered to be an inducer of cellular senescence [21]. Cellular senescence is suggested to arrest the cells in G1/S phase of the cell cycle. On one hand, this could be beneficial if cancer cells stop proliferating [8, 22]. On the other hand, the beneficiary effect might be counteracted by the ability of senescent cells to secrete a senescence-associated secretory phenotype (SASP), which contains cytokines and chemokines that may promote the development of cancer in later stage of life or treatment [22, 23]. In general, growth inhibition mediated by AR antagonists is associated with induction of cellular senescence [5, 6, 24] and is suggested as a cellular tumor suppressive pathway [8].

Senescent cells occur during tumorigenesis in premalignant tumors [9, 22]. It is suggested that malignant tumor cells evade the pathways of cellular senescence and apoptosis to become more aggressive and malignant [9]. Thus, reenforcing cellular senescence in malignant tumors could therefore be beneficial. Interestingly, various antagonists of AR induce the cellular pathway of senescence including atraric acid, spironolactone derivatives, compound C28, ENZ and ODM [5, 6, 24, 25]. These findings suggest that the AR is not completely inactivated by treatment with AR antagonists but retains the senescence inducing signaling and tumor suppressive functions perhaps by non-direct DNA binding through interaction with other factors.

Interestingly as shown here, IL-23 reduced the level of AR antagonist-induced cellular senescence in both C4-2 and 22Rv1, model cell lines for CRPC. This confirms that IL-23 might serve as a potential drug target [10]. Notably, the senescence-suppressive effect of IL-23 was not observed by IL-23 alone but in the presence of AR antagonists. Moreover, the data indicate that IL-23 senescence-suppressive function was preferentially observed for CRPC cell lines but not for the androgen-sensitive LNCaP cells. It is unclear what alternative pathway is used by IL-23 in comparison with C4-2 cells as the castration-resistant derivative of LNCaP cells. Since IL-23 also reduced the antagonist-induced cellular senescence level of 22Rv1, it suggests that IL-23 affects AR signaling preferentially in CRPC cells. This may also indicate that IL-23 counteracts ENZ and ODM signaling, and therefore less cancer cells are rendered senescent. Thus, the data provide the basis for further analyses of how IL-23 inhibits the induction of cellular senescence in CRPC induced by AR antagonists.

## Electronic Supplementary Material

ESM 1 (PDF 373 kb).

## Data Availability

All data generated or analyzed during this study are included in this published article and its supplementary information files.
