# Supplementary material for: Interleukin-23 Represses the Level of Cell Senescence Induced by the Androgen Receptor Antagonists Enzalutamide and Darolutamide in Castration-Resistant Prostate Cancer Cells
Source: Horm Cancer. 2020 Jun 20;11(3-4):182–90. doi: 10.1007/s12672-020-00391-5 (PMC7335377; doi:10.1007/s12672-020-00391-5)

Supplementary Figure 1

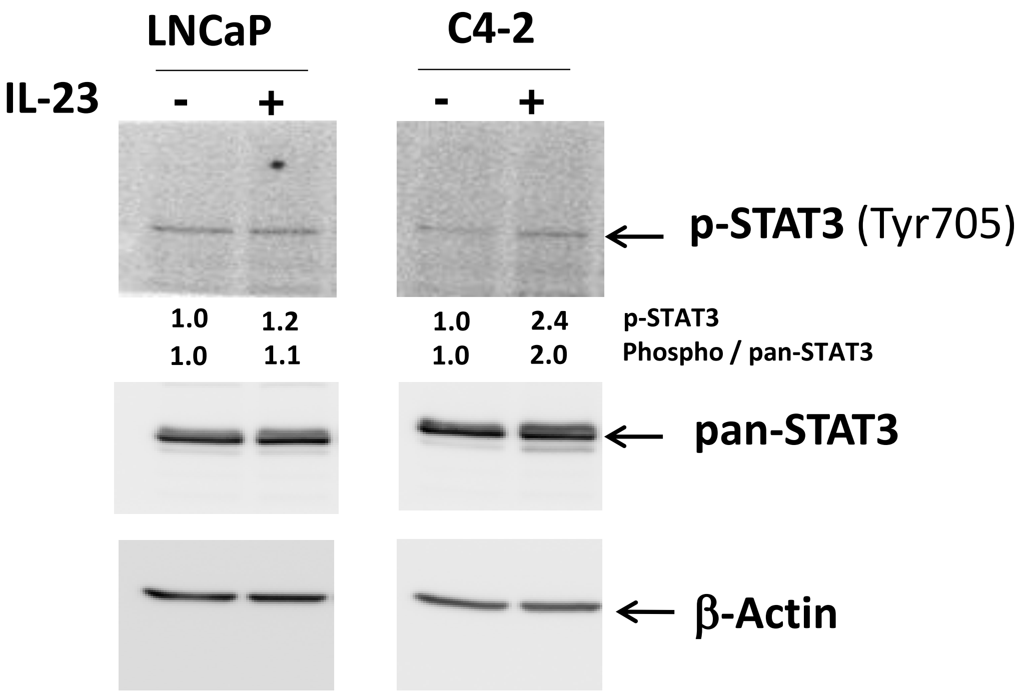

Fig. S1 IL-23 enhances levels of phospho-STAT3 (p-STAT3)
Western blot of LNCaP and C4-2 extracts treated with IL-23 reveal about a two fold increase of p-STAT3 (Tyr705) in C4-2 cells. Band intensities were normalized to β-Actin. The numbers indicate the relative p-STAT3 levels and the ratio between p-STAT3 and pan-STAT3.

Supplementary Figure 2

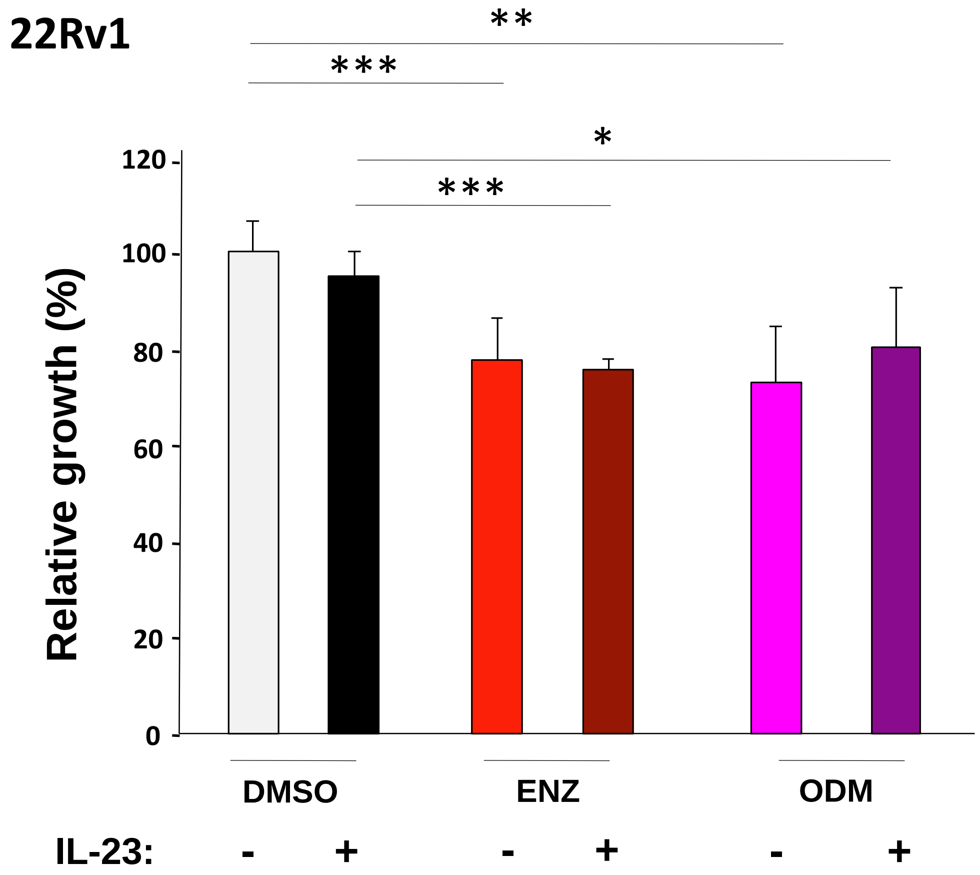

Fig. S2 AR antagonists suppress proliferation of CRPC 22Rv1 cells.
Same experimental setup as in Fig. 1b and c except using 22Rv1 cells.

Supplementary Figure 3

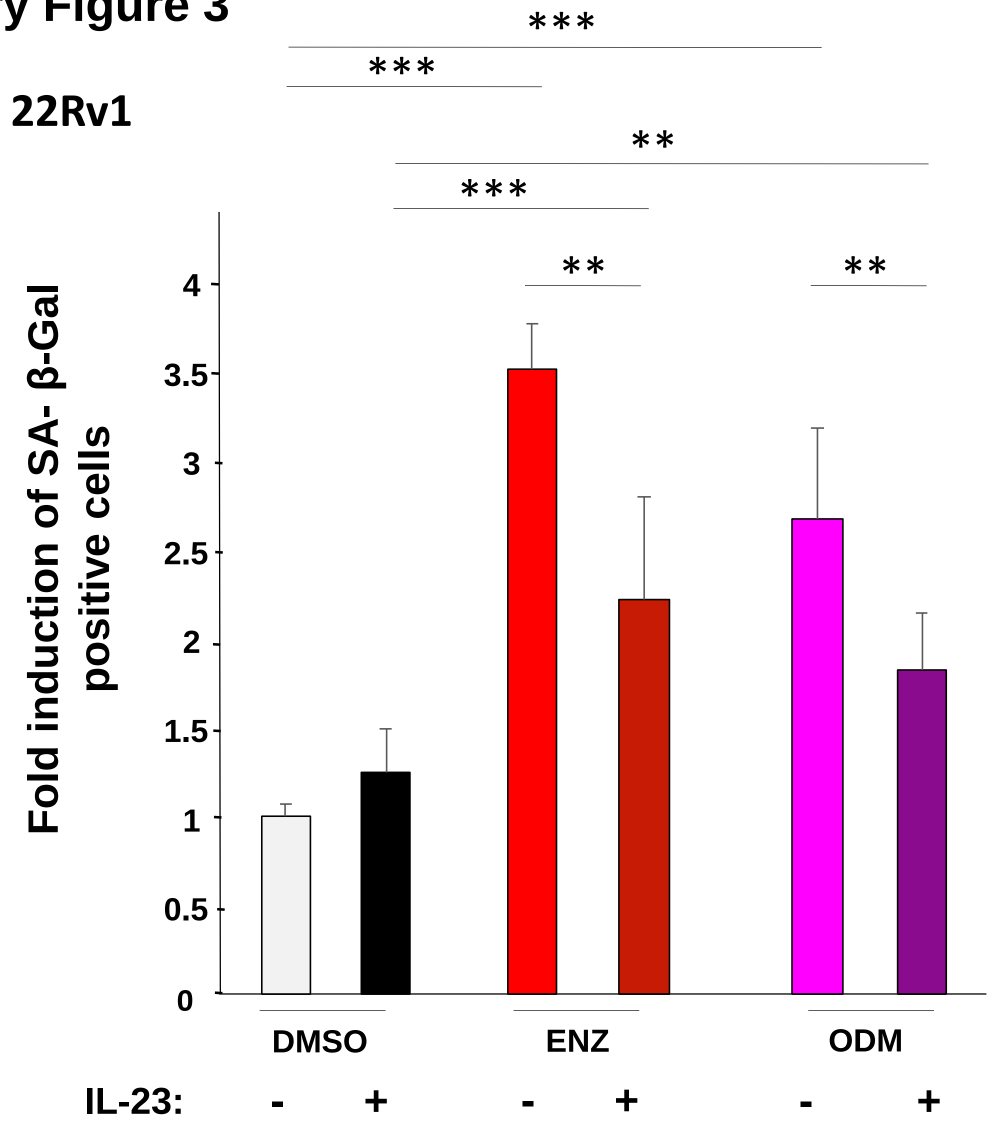

Fig. S3 IL-23 reduces AR antagonist-induced cellular senescence in 22Rv1 cells.
Same experimental setup as in Fig. 3 except using 22Rv1 cells.

Supplementary Figure 4

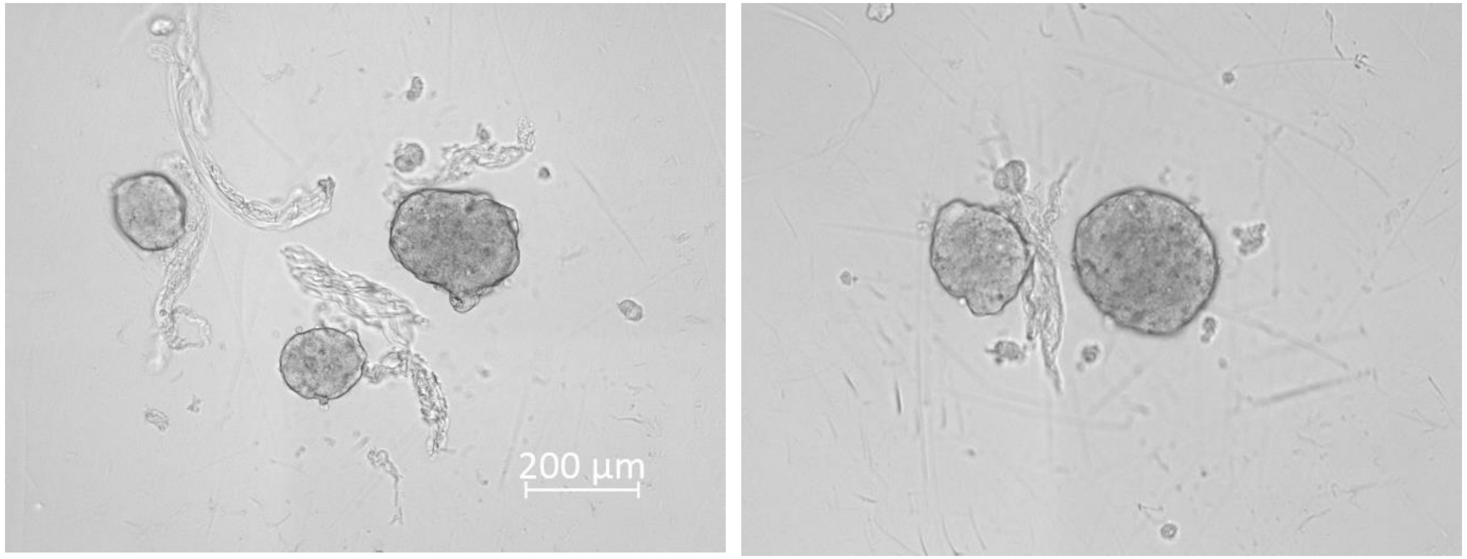

Fig. S4 Lack of proper 22Rv1 spheroid formation.
Same experimental setup as in Fig. 5 except using 22Rv1 cells. Data suggest that 22Rv1 form multiple small spheroids in contrast to C4-2 cells under same conditions.

Supplementary Figure 5

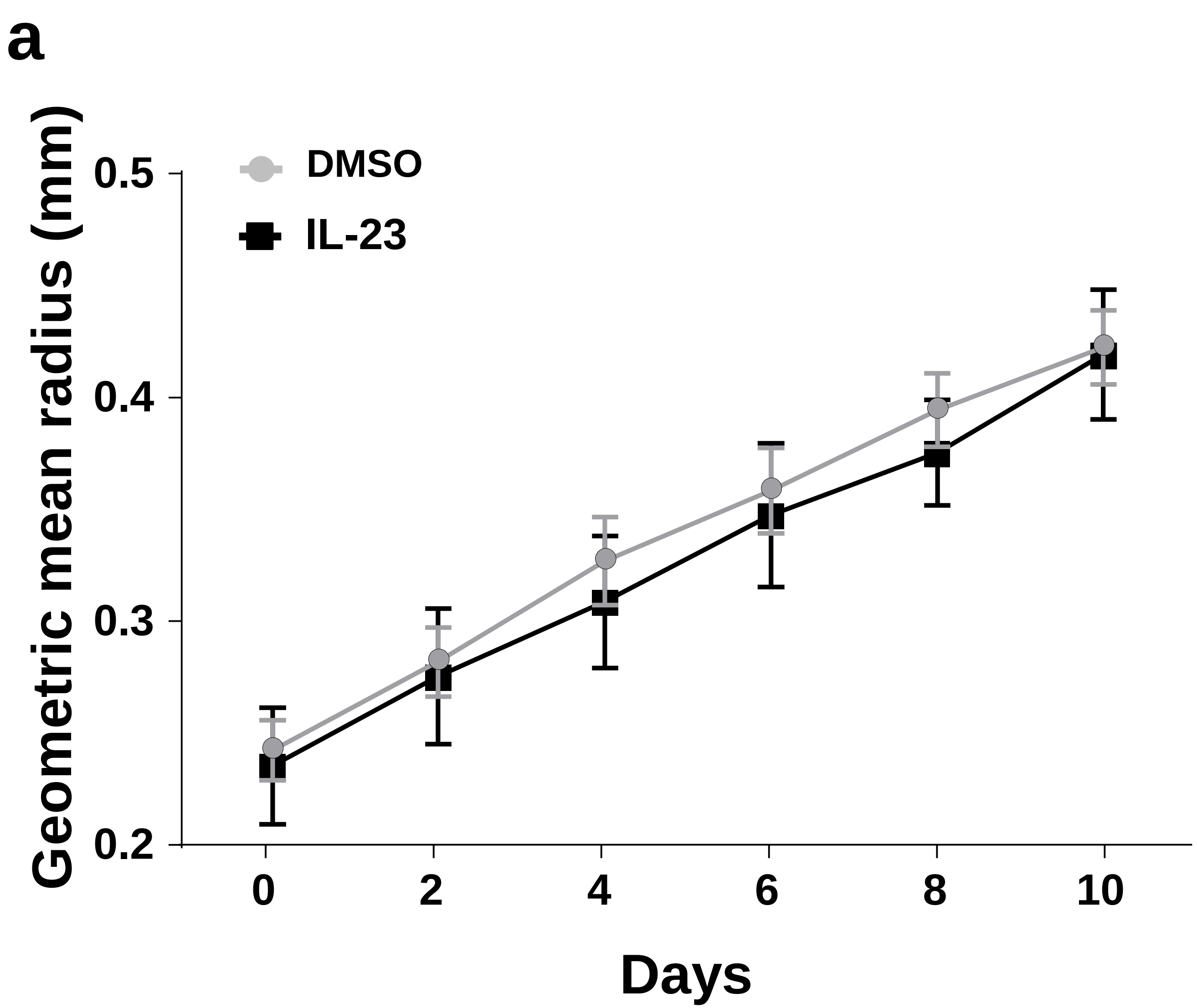

Fig. S5 Change of mean radius of spheroids.
Same experimental setup as in Fig. 5 depicting the measured mean radius. (a) control treatment, (b) ENZ and (c) ODM each treatment with and without IL-23

Supplementary Figure 5

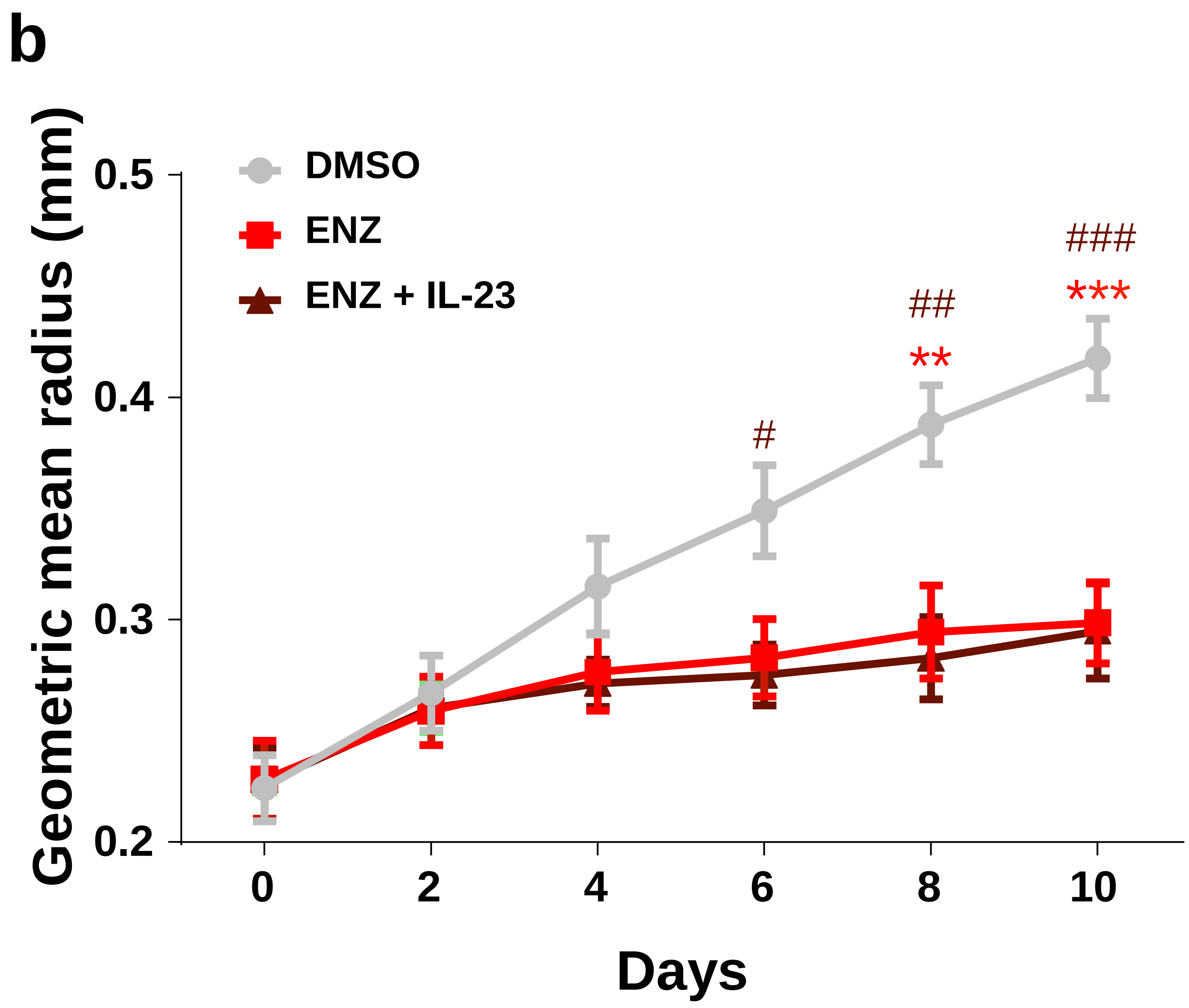

Supplementary Figure 5

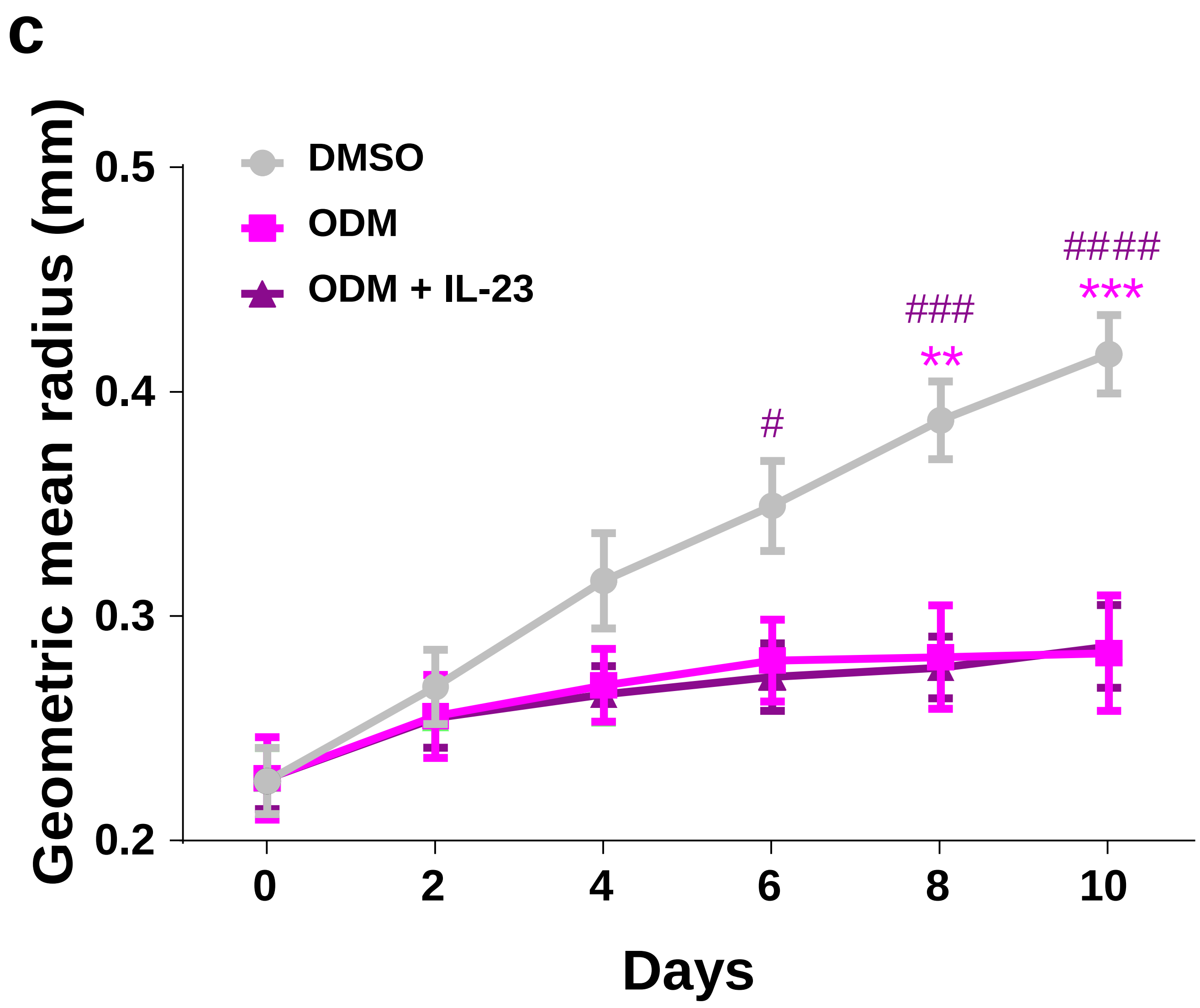

Supplement: Supplementary file 1 — (PDF 373 kb). [file 12672_2020_391_MOESM1_ESM.pdf]
